# Incarcerated Incisional Hernia: An Unusual Presentation of Metastatic Endometrial Carcinoma

**DOI:** 10.7759/cureus.71497

**Published:** 2024-10-14

**Authors:** Mang Ning Ong, Guo Hou Loo, Guhan Muthkumaran, Suria Hayati Md Pauzi, Nik Ritza Kosai

**Affiliations:** 1 Upper GI and Metabolic Surgery Unit, Department of Surgery, Hospital Canselor Tuanku Muhriz, Universiti Kebangsaan Malaysia, Kuala Lumpur, MYS; 2 Department of Pathology, Hospital Canselor Tuanku Muhriz, Universiti Kebangsaan Malaysia, Kuala Lumpur, MYS

**Keywords:** endometrial serous carcinoma, gynaecological malignancy, incarcerated incisional hernia, incisional ventral hernia, strangulated ventral hernia

## Abstract

Abdominal wall hernia is a common condition seen in the clinical practice of surgery. However, malignant tumors in the hernia sac are rare and there are limited studies on this subject. We report a case of a 77-year-old female who presented with generalized abdominal pain and vomiting. She was treated for an incarcerated incisional hernia and underwent an exploratory laparotomy, which showed a multiseptated incisional hernia sac. Histopathological examination revealed a metastatic endometrial serous carcinoma (ESC). ESC is an aggressive variant associated with poor prognosis, characterized by metastasis and extrauterine spread. Its treatment mainly involves a multidisciplinary approach, including surgical treatment and chemoradiotherapy. This report highlights the importance of considering malignant tumors in the differential diagnosis of hernia sac contents. Raising awareness among healthcare professionals and the general public can aid in the prompt diagnosis, appropriate treatment, and improved outcomes for individuals with such rare presentations.

## Introduction

Abdominal wall hernia is a condition commonly encountered in the clinical practice of surgery. Incisional hernias are abdominal wall defects occurring over a previous surgical site or incision. They can present as a hernia with all the typical components, including a defect, a sac, and herniated contents. Alternatively, it can manifest as a weakness in the abdominal wall, featuring a shallow sac with intermittent bulging of contents. The rate for incisional hernia varies with the type of procedures performed, along with different patient-related factors. While it typically ranges from 3 to 13%, it was reported to be as high as 35% in midline laparotomies in one study [1,2].

While incisional hernias can remain asymptomatic, they may result in complications that lead to significant issues like pain, bowel obstruction, incarceration, and strangulation, often requiring surgical intervention for treatment [1]. The contents of an incisional hernia vary depending on the location and size. The contents commonly include bowel loops, omentum, preperitoneal fats, and, in rare cases, bladder, gynecological organs, and even malignancy. However, malignant tumors in the hernia sac are rare and there are limited studies on this subject.

Tumors in the hernia sac usually entail a secondary involvement of a primary malignancy or can also be due to metastasis [3]. The existing literature primarily consists of isolated case reports or limited case series concerning malignant tumors within hernia sacs [3,4]. The incidence of malignant tumors in hernia sacs is low, with studies reporting rates of 0.14-0.59% [3]. The presence of malignancy in a hernia sac can be an incidental finding during hernia repair surgery, and it is often associated with advanced disease and poor prognosis. Female patients have been reported to be more likely to have gynecological primary tumors, particularly serous ovarian carcinoma, whereas male patients more commonly have gastrointestinal or pancreaticobiliary malignancies involving the hernia sac [4].

We report a case of a 77-year-old female patient who presented with generalized abdominal pain and vomiting. She was treated for an incarcerated incisional hernia and underwent an exploratory laparotomy, which showed a multiseptated incisional hernia sac. Histopathological examination revealed a metastatic endometrial serous carcinoma (ESC).

## Case presentation

A 77-year-old female with a BMI of 54 kg/m^2^ with underlying type 2 diabetes mellitus, hypertension, and asthma presented to the emergency department with generalized abdominal pain and vomiting for one day. The patient had a history of paraumbilical hernia repair performed more than 25 years ago. She had been having abdominal swelling over the previous repair site for more than 10 years, which had been irreducible for the past five years. There had been intermittent abdominal discomfort for three months, which had worsened over the past week.

**Figure 1:**
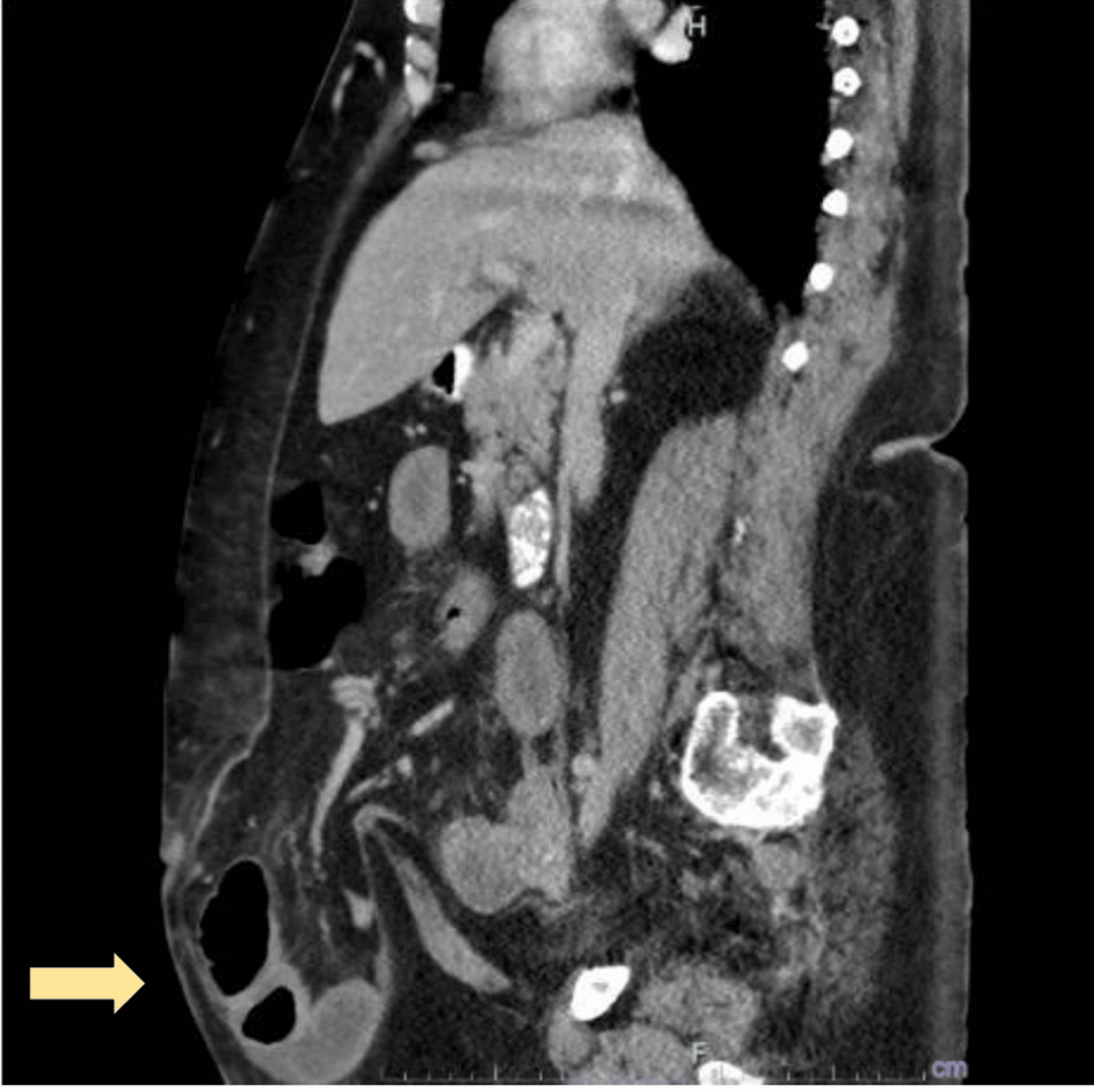
CT abdomen (coronal view) The image shows incisional hernia with thickened hernia sac with small bowel content (yellow arrow) CT: computed tomography

**Figure 2:**
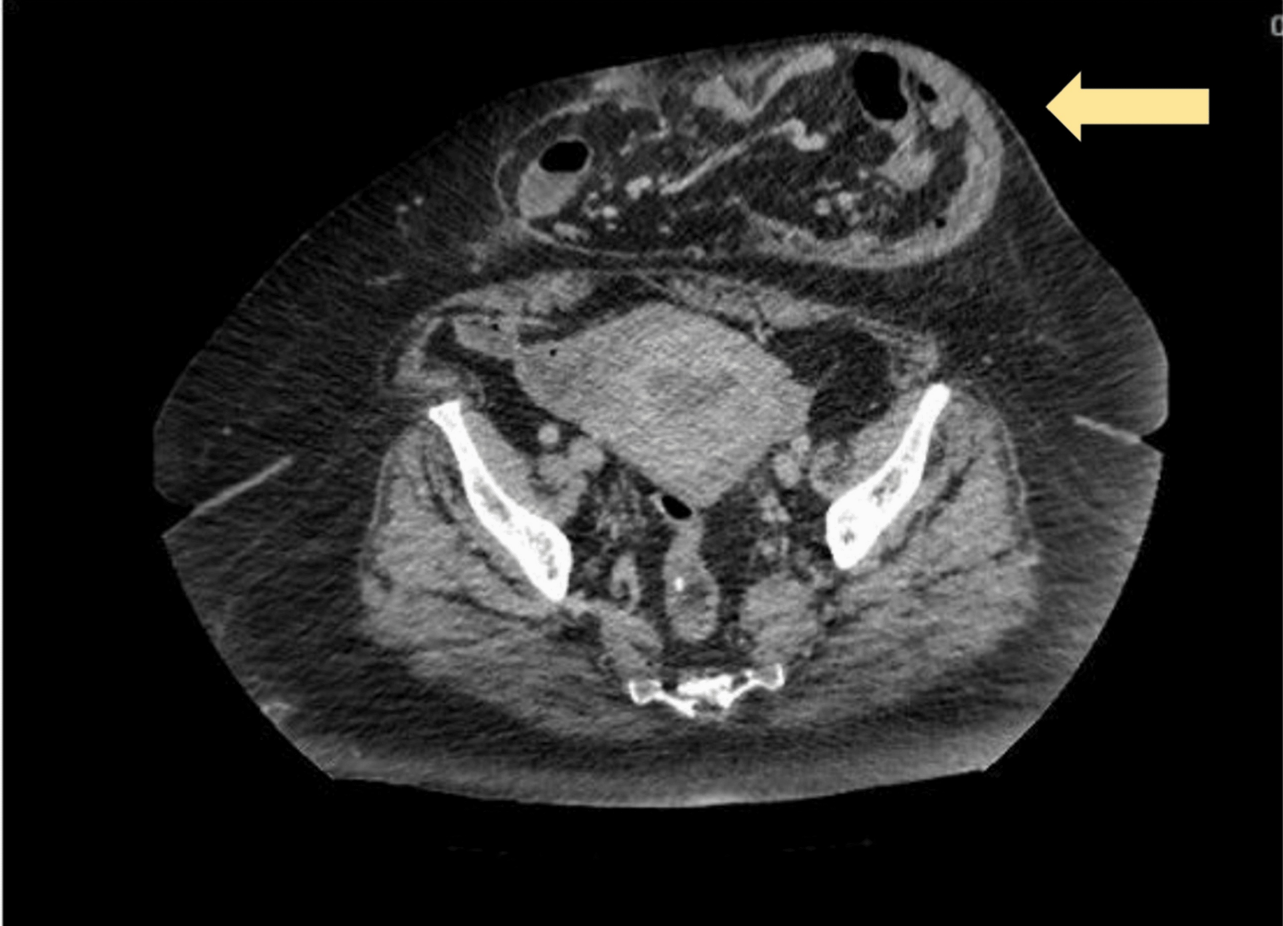
CT abdomen and pelvis (axial view) at the level of the pelvis The image shows the incisional hernia with thickened hernia sac (yellow arrow) CT: computed tomography

Clinical examination revealed a large, tender, and irreducible incisional hernia over her central abdomen measuring about 25 x 17 cm. An urgent CT of the abdomen and pelvis revealed a large lobulated anterior abdominal wall hernia with small bowel content suggestive of strangulation (Figures 1-2). A provisional diagnosis of a strangulated incisional hernia was made and she was brought to the theatre for an exploratory laparotomy.

**Figure 3:**
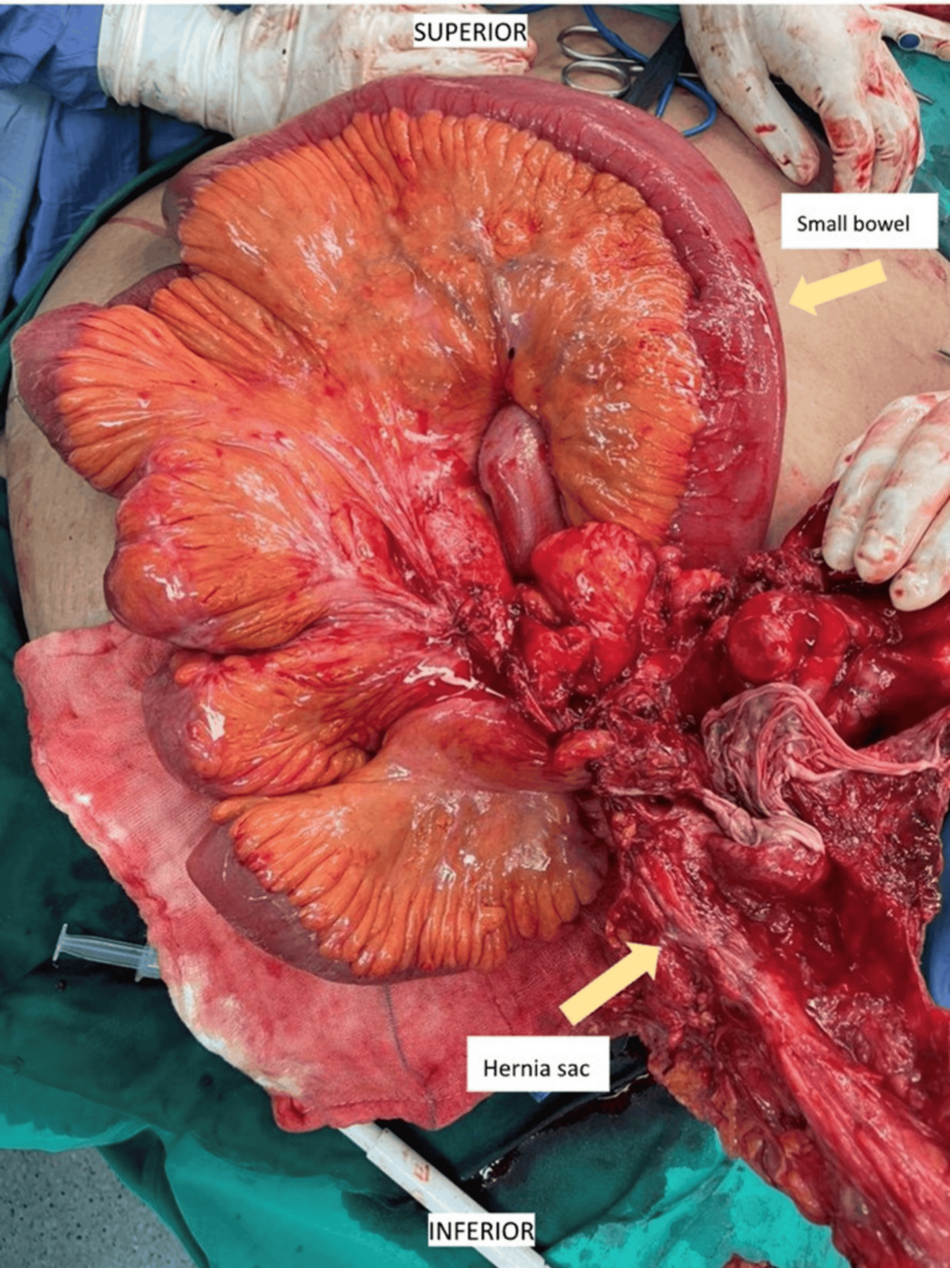
Intraoperative image showing loops of small bowel with clumped omentum, with part of the fibrosed hernia sac adhered with small bowel

**Figure 4:**
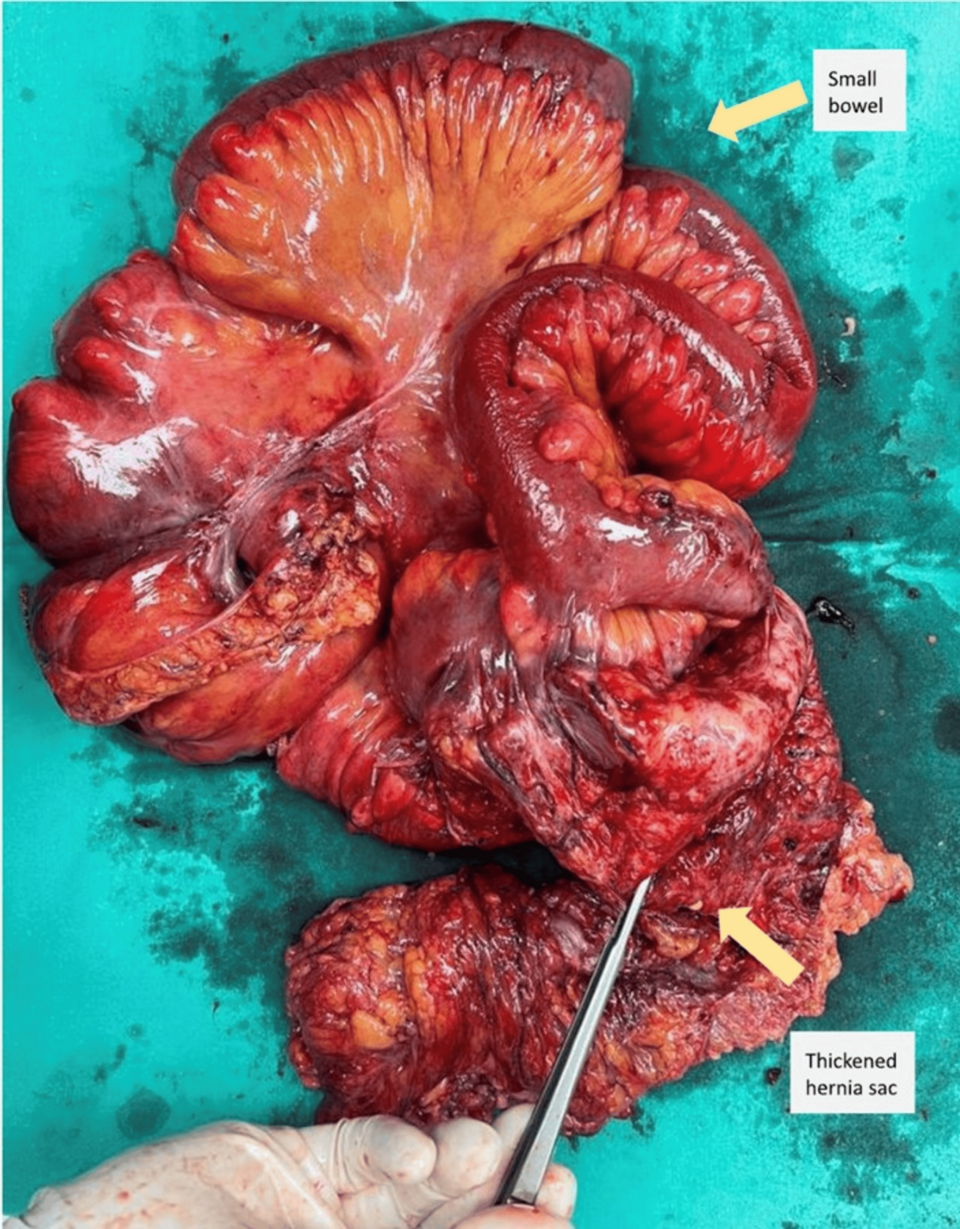
Specimen image of the en-bloc resected small bowel (100 cm in length) with thickened hernia sac

Intraoperatively, a multiseptated hernia sac measuring about 20 x 25 cm containing clumped omentum as well as small and large bowel was seen (Figures 3-4). Part of the terminal ileum was densely adhered to the fibrosed hernia sac, and segmental small bowel en bloc resection along with the hernia sac was performed. Abdominal closure was performed using continuous black double-strand monofilament polyamide loop suture with the reinforced tension line technique. 

**Figure 5:**
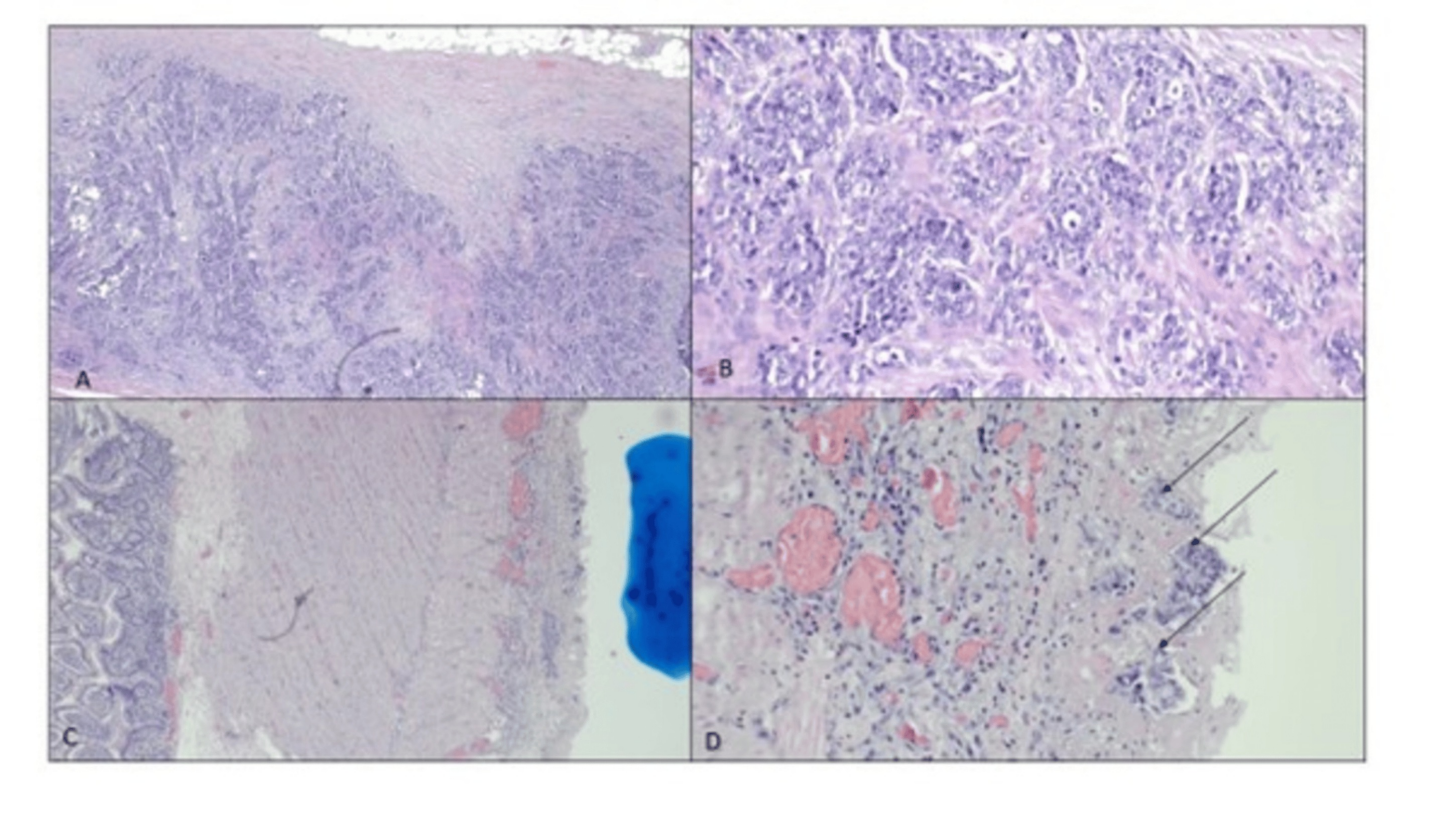
Histopathological images (A) Malignant cell infiltration is observed within the fibrous adhesion (H&E, 4x). (B) The malignant cells are arranged in clusters with occasional lumen seen exhibiting enlarged hyperchromatic nuclei and small nucleoli (H&E, 20x). (C) Tumor deposits on the serosal surface of the small bowel (blue-inked area) (H&E, 4x). (D) The malignant cells show similar cytomorphology as in (A) (black arrow) (H&E, 20x)

**Figure 6:**
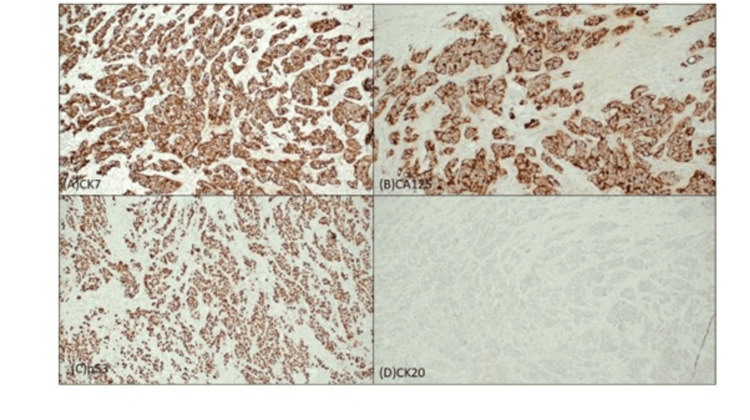
Immunochemistry staining slide images (A) The tumor cells are immunopositive positive to CK7 (10x), (B) CA125 (10x), and (C) p53 (10x). (D) CK20 stain is negative (10x)

Histopathological examination revealed that there was no malignancy in the resected small bowel. However, the area between the fibrous adhesion and the small bowel showed foci of tumors that did not infiltrate the small bowel. Immunohistochemistry staining showed diffuse, strong positivity toward CK7, CA125, and p53 (Figures 5-6). These findings were compatible with a possible metastatic carcinoma from a gynecological primary.

**Figure 7:**
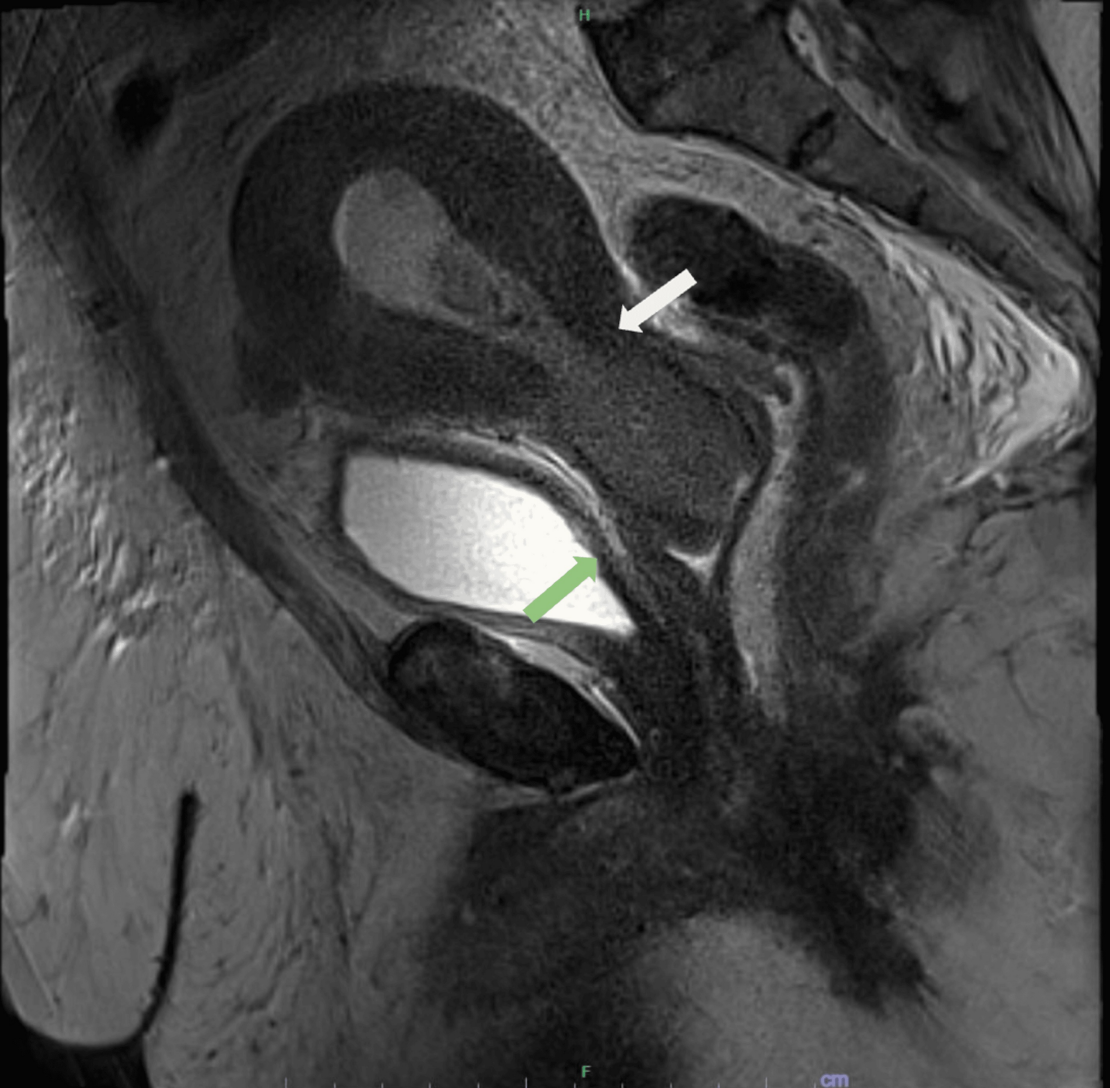
Sagittal view of T2-contrasted MRI of the pelvis The image shows cervical endometrial malignancy with cervical stroma invasion (white arrow) along with thickened peritoneum (green arrow) MRI: magnetic resonance imaging

An endometrial pipelle was performed, and it demonstrated serous carcinoma. Serum CA-125 was elevated at 105 U/ml (normal range: 0-35 U/ml) and an MRI pelvis demonstrated cervical-endometrial malignancy with cervical stromal invasion and hematometra (Figure 7). There was also evidence of peritoneal metastasis. After a multidisciplinary meeting, a diagnosis of stage 4 ESC with peritoneal metastasis was made. The patient subsequently received palliative chemotherapy (paclitaxel and carboplatin three weekly) but unfortunately succumbed to her disease seven months later.

## Discussion

Endometrial carcinoma (EC) is one of the most common gynecological malignancies, usually seen in elderly, multiparous, and post-menopausal women. ESC is an aggressive variant associated with poor prognosis, in which metastasis and extrauterine spread are commonly seen. Up to about two-thirds of patients have distant metastasis during the time of diagnosis with omentum being the most common site. There is a strong association between positive p53 with ESC, as up to 84% of patients were found to have positive p53 [5]

Hernias of the abdominal wall are one of the common conditions seen, and they can be found in up to 1.7% of patients of all ages and 4% of those above 45 years of age [6]. However, malignant tumors within the hernia sac are usually discovered incidentally and not commonly seen. In a study by Seçinti et al., 10 out of 455 hernia sacs were noted to be positive for malignancy [7]. Out of all the malignancies, colorectal metastasis was the most commonly seen variant, as reported by Zhang et al. Other common primaries include hepatobiliary and gynecological malignancies. Female patients were also reported to have a predisposition to gynecological primary, particularly ovarian serous carcinoma [4].

Endometrial malignancy commonly presents as postmenopausal vaginal bleeding. Other symptoms of the condition include abdominal pain, feeling an abdominal mass, increasing abdominal size, or bloatedness. There are scarce studies in the literature reporting ovarian malignancy presenting as an obstructed hernia. Hernias can usually be diagnosed on clinical examination alone. Gately et al. have emphasized the importance of investigating recurrent hernia in middle-aged women as the suspicion of causative intraabdominal pathology should be considered [8]. There have also been cases reporting incidental findings of EC presenting as umbilical metastasis - Sister Mary Joseph’s nodule (SMJN) - which is usually a reflection of a widespread malignancy. Patients presenting with SMJN typically have a poor prognosis [9].

Diagnosis of EC usually requires a series of investigations: blood tests such as CA-125, microscopy-based tests including pap smear, hysteroscopy, cystoscopy, and dilatation and curettage. Most importantly, examining the endometrial biopsy specimen is required as it is effective and sensitive in diagnosing EC [10]. In cases similar to ours, radiological imaging such as ultrasound scan, CT, and MRI would be helpful and play a key role in the diagnosis. The staging of EC is based on the International Federation of Gynaecology and Obstetrics (FIGO) guidelines. The prognosis for ESC is poorer compared to other variants. The five-year overall survival rate for stage IV disease, as described in our patient, is only 33% [11].

Treatment for EC mainly involves a multidisciplinary approach, which includes surgical treatment followed by chemoradiotherapy. Surgical treatment depends on the stage of the disease; however, the treatment of choice would be a total hysterectomy, bilateral salpingo-oophorectomy, bilateral pelvic lymphadenectomy, systematic para-aortic lymphadenectomy (with/without omentectomy), and peritoneal cytology. Subsequent platinum/taxane-based chemotherapy is recommended. Radiotherapy is used to achieve a better clinical outcome [12]. Other treatment options include anti-HER2/neu antibody - trastuzumab. However, studies have shown no significant improvement in the treatment with trastuzumab alone versus that combined with chemotherapy, which has shown better clinical outcomes [13].

## Conclusions

Malignant tumors within hernia sacs are rare but can pose diagnostic and therapeutic challenges. This report underscores the importance of a comprehensive approach involving imaging, biopsy, and collaboration between surgical and oncological specialties for effective management. The decision to send a hernia sac for routine pathology is often based on individual surgeon practices and institutional guidelines, and subtle lesions may be overlooked on gross examination. This report highlights the significance of considering malignant tumors in the differential diagnosis of hernia sac contents. Raising awareness among healthcare professionals and the general public can help with prompt diagnosis, appropriate treatment, and improved outcomes for individuals with such rare presentations. Further research is warranted to enhance our understanding of the incidence, risk factors, and optimal management of malignant tumors in hernia sacs.

## References

[1] (2007). SSAT patient care guidelines. Surgical repair of incisional hernias. J Gastrointest Surg.

[2] Stabilini C, Garcia-Urena MA, Berrevoet F (2022). An evidence map and synthesis review with meta-analysis on the risk of incisional hernia in colorectal surgery with standard closure. Hernia.

[3] Val-Bernal JF, Mayorga M, Fernández FA, Val D, Sánchez R (2014). Malignant epithelial tumors observed in hernia sacs. Hernia.

[4] Zhang D, Yang Q, Katerji R, Drage MG, Huber A, Liao X (2020). Malignant tumors in hernia sac: clinicopathological and immunohistochemical studies of 21 cases at a single institution. Pathol Int.

[5] Agarwal A, Yadav S, Dusane R, Menon S, Rekhi B, Deodhar KK (2022). Endometrial serous carcinoma: a retrospective review of histological features & their clinicopathological association with disease-free survival & overall survival. Indian J Med Res.

[6] Jenkins JT, O’Dwyer PJ (2008). Inguinal hernias. BMJ.

[7] Seçinti İE, Gürsoy D, Özgür T, Hakverdi S, Doğan E, Temiz M (2022). Surprise in hernia sacs: malignant tumor metastasis. J Surg Med.

[8] Gately RP, Concannon ES, Hogan A, Ryan RS, O'Leary M, Barry K (2012). Recurrent femoral hernia and associated ovarian pathology. BMJ Case Rep.

[9] Piura B, Meirovitz M, Bayme M, Shaco-Levy R (2006). Sister Mary Joseph's nodule originating from endometrial carcinoma incidentally detected during surgery for an umbilical hernia: a case report. Arch Gynecol Obstet.

[10] Zhang L, Kwan SY, Wong KK, Solaman PT, Lu KH, Mok SC (2020). Pathogenesis and clinical management of uterine serous carcinoma. Cancers (Basel).

[11] Ferriss JS, Erickson BK, Shih IM, Fader AN (2021). Uterine serous carcinoma: key advances and novel treatment approaches. Int J Gynecol Cancer.

[12] DeNardis SA, Holloway RW, Bigsby GE 4th, Pikaart DP, Ahmad S, Finkler NJ (2008). Robotically assisted laparoscopic hysterectomy versus total abdominal hysterectomy and lymphadenectomy for endometrial cancer. Gynecol Oncol.

[13] Fader AN, Roque DM, Siegel E (2018). Randomized phase II trial of carboplatin-paclitaxel versus carboplatin-paclitaxel-trastuzumab in uterine serous carcinomas that overexpress human epidermal growth factor receptor 2/neu. J Clin Oncol.

